# Phenotypic alteration of low-density granulocytes in people with pulmonary post-acute sequelae of SARS-CoV-2 infection

**DOI:** 10.3389/fimmu.2022.1076724

**Published:** 2022-12-15

**Authors:** Logan S. Dean, Gehan Devendra, Boonyanudh Jiyarom, Natalie Subia, Michelle D. Tallquist, Vivek R. Nerurkar, Sandra P. Chang, Dominic C. Chow, Cecilia M. Shikuma, Juwon Park

**Affiliations:** ^1^ Hawaii Center for AIDS, John A. Burns School of Medicine, University of Hawai’i at Manoa, Honolulu, HI, United States; ^2^ Department of Tropical Medicine, Medical Microbiology, and Pharmacology, John A. Burns School Medicine, University of Hawai’i at Manoa, Honolulu, HI, United States; ^3^ Department of Pulmonary and Critical Care, Queen’s Medical Center, Honolulu, HI, United States; ^4^ Department of Medicine, John A. Burns School of Medicine, University of Hawai’i at Manoa, Honolulu, HI, United States; ^5^ Center for Cardiovascular Research, John A. Burns School of Medicine, University of Hawai’i at Mānoa, Honolulu, HI, United States

**Keywords:** SARS-CoV-2, long-COVID, post-acute sequalae of SARS-CoV-2 infection, pulmonary sequelae, low-density granulocyte, neutrophil extracellular traps, platelets, platelet-neutrophil aggregates

## Abstract

**Background:**

Low-density granulocytes (LDGs) are a distinct subset of neutrophils whose increased abundance is associated with the severity of COVID-19. However, the long-term effects of severe acute respiratory syndrome coronavirus 2 (SARS-CoV-2) infection on LDG levels and phenotypic alteration remain unexplored.

**Methods:**

Using participants naïve to SARS-CoV-2 (NP), infected with SARS-CoV-2 with no residual symptoms (NRS), and infected with SARS-CoV-2 with chronic pulmonary symptoms (PPASC), we compared LDG levels and their phenotype by measuring the expression of markers for activation, maturation, and neutrophil extracellular trap (NET) formation using flow cytometry.

**Results:**

The number of LDGs was elevated in PPASC compared to NP. Individuals infected with SARS-CoV-2 (NRS and PPASC) demonstrated increased CD10^+^ and CD16^hi^ subset counts of LDGs compared to NP group. Further characterization of LDGs demonstrated that LDGs from COVID-19 convalescents (PPASC and NRS) displayed increased markers of NET forming ability and aggregation with platelets compared to LDGs from NP, but no differences were observed between PPASC and NRS.

**Conclusions:**

Our data from a small cohort study demonstrates that mature neutrophils with a heightened activation phenotype remain in circulation long after initial SARS-CoV-2 infection. Persistent elevation of markers for neutrophil activation and NET formation on LDGs, as well as an enhanced proclivity for platelet-neutrophil aggregation (PNA) formation in COVID-19 convalescent individuals may be associated with PPASC prognosis and development.

## Introduction

Since December 2019, severe acute respiratory syndrome coronavirus 2 (SARS-CoV-2) has circulated worldwide, infecting over 600 million people globally while being responsible for over 6 million deaths (1). Although most people with COVID-19 infection recover within a few weeks, almost a third of survivors suffer from persistent symptoms, so called “Long COVID”, otherwise known as Post-Acute Sequelae of SARS-CoV-2 Infection (PASC) (2). PASC symptoms are highly variable and sustained for more than 30 days after initial COVID-19 infection (3). Development of Pulmonary PASC (PPASC), which includes persistent dyspnea and chronic cough, is not limited to recovered patients with prior hospitalizations or severe acute SARS-CoV-2 infection (4). Although studies have been initiated to identify factors associated with developing PPASC, mechanisms responsible for PPASC pathology remain unknown.

A growing body of literature implicates neutrophils as drivers of the hyperinflammatory state observed during acute COVID-19 (5–8). Increased neutrophil numbers are consistently reported during hospitalization due to COVID-19, and the neutrophil to lymphocyte ratio (NLR) is considered an independent risk factor for mortality among hospitalized COVID-19 patients (7). Moreover, Neutrophil Extracellular Traps (NETs), one of neutrophils’ defense mechanisms, is known to contribute to the maladaptive immune response *via* promotion of a hyperinflammatory state and *via* initiation of thrombotic events during COVID-19 (9–11). Histological examination of lung autopsies from severe COVID-19 patients reveal abundant NETs associated with microthrombi within alveolar capillaries (12). The combined evidence demonstrates that excessive neutrophils and NET production is associated with increased disease severity, pathophysiology, and poor clinical outcomes in COVID-19 (13).

Low-density granulocytes (LDGs) are a subpopulation of neutrophils that co-exist within peripheral blood mononuclear cell (PBMC) fractions (13). These cells demonstrate an enhanced ability to form NETs and are known to contribute to immune-mediated pathology through proinflammatory cytokine production and mediation of endothelial cell death (14–16). In the context of acute COVID-19 infection, frequencies of LDGs in individuals with mild to severe COVID-19 are increased compared to healthy controls (17, 18). Additionally, their frequencies are positively correlated with markers for granulocyte recruitment and activation (14, 19–21). Although elevated levels of LDGs and phenotypic activation are thought to play an important role in disease severity in COVID-19 patients, their circulating levels and functional contributions to long-term effects of COVID-19 and PPASC development are currently unknown.

In this study, we characterized LDG populations in individuals up to fourteen months following SARS-CoV-2 infection, alongside comparator groups, with and without, residual pulmonary symptoms. Our findings demonstrated that an altered LDG phenotype and increased NET and PNA formation are associated with prior SARS-CoV-2 infection and further enhanced in individuals with PPASC.

## Methods

### Study subjects and specimens selection

The “COVID-19 Infection in Hawaii” study is a cross sectional study of PASC complications in which participants were recruited from the state’s major tertiary care hospital. Participants completed written informed-consent documents and questionnaires regarding demographics, relevant medical history, and acute and/or lingering COVID-19 symptoms. PPASC patients were identified based on pulmonary function tests specifically a reduced predicted diffusing capacity for carbon monoxide (DLCOc, <80%) value, corrected for the patientʻs hemoglobin. Comparitor study groups included patients fully recovered from SARS-CoV-2 infection with no residual symptoms >30 days after acute infection (NRS; n=10). Normal participants were selected from an existing HIV cohort study and who were HIV negative (NP; n=12). These groups were age- and gender-matched to the PPASC group for data analysis. The study was approved by the Queens Medical Center Institutional Review Committee with the University of Hawaii IRB ceding authority (IRB#: RA-2020-053).

### Plasma and PBMC isolation

Whole blood was collected in EDTA tubes by venipuncture and processed within 1 hour of collection based on previously published methods (22). Whole blood was centrifuged, plasma collected and cryopreserved at −80°C until downstream analysis was done. A separate aliquot of the whole blood was diluted with an equal volume of PBS overlayed onto Ficoll-Paque Plus (GE Healthcare Biosciences, Piscataway, NJ) according to manufacturer’s protocol. PBMC were aspirated off the Ficoll interphase, red blood cells lysed, and then washed twice in PBS supplemented with 1% Fetal Bovine Serum (FBS). Cells were then counted, viability determined, and cryopreserved at −80°C until further analysis.

### Quantification of cell-free DNA

Circulating cell-free DNA (cfDNA) in the plasma was quantified using the Quant-iT™ PicoGreen™ dsDNA Assay-Kit (ThermoScientific, P11496) according to the manufacturer’s instructions. Briefly, samples were diluted 1:20 in TE buffer and Quant-iT™ PicoGreen™ dsDNA reagent was added in a 1:1 ratio. After incubation for 5 min at RT in the dark, the fluorescence was read in a VICTOR3 plate reader (Perkin Elmer). The concentration of the circulating cell-free DNA was calculated using the standard provided by the kit.

### Enzyme-linked immunosorbent assay for NETs

#### Quantification of citrullinated histone H3

Citrullinated histone H3 (CitH3) was quantified in plasma using the enzyme-linked immunosorbent assay according to the manufacturer’s instructions (Cayman, 501620). 100 uL of the standard and diluted sample (1:10 in 1x assay buffer) were added to the 96-well pre-coated plate with CitH3 antibodies. After incubation for 2 hours at RT on an orbital shaker, the plate was washed five times. Then, 100 uL of the HRP conjugate working solution was added to each well, covered, incubated for one hour at RT on an orbital shaker, and then washed five times to eliminate the excess. Lastly, 100 uL of TMB substrate solution was added to each well, covered, and incubated for 30 minutes at RT on an orbital shaker. Immediately after, 100 uL of HRP stop solution was added to each well. The absorbance was measured at 450nm using the microplate reader (The SpectraMax® M3, Molecular Devices, San Jose, CA). Concentrations were calculated for each sample depending on the generated standard curve from the given standards.

### Flow cytometric analysis

Typically, 1-2x10^6^ PBMC were incubated with Fixable Viability Dye eFluor 506 (Ebiosciences, 1:1000) at 4°C for 30 minutes, followed by addition of Human TruStain FcX (BioLegend, San Diego, CA, 1:200) in flow buffer (HBSS supplemented with 1% BSA) at room temperature (RT) for 15 minutes. Subsequently, cells were stained with the titrated fluorophore-conjugated primary antibody cocktail (**Table S1**) at RT for 30 minutes and then washed twice with ice-cold flow buffer. For the intracellular staining, cells were resuspended in 250 mL of BD Cytofix/Cytoperm (BD Technologies, East Rutherford, NJ) for 30 minutes at 4°C and then incubated with the titrated primary anti-Myeloperoxidase (MPO)-PE and primary citrullinated histone H3 (citH3) antibody (Abcam, Waltham, MA) for 30 minutes at 4°C, protected from light. Allophycocyanin (APC)-conjugated anti-goat secondary antibody (Invitrogen, Waltham, MA) at 1:500 dilution was added for labeling the anti-citH3 primary antibody. Samples were then washed twice with ice-cold flow buffer and resuspended to 800 mL of flow buffer for acquisition. Samples were acquired on a Attune NxT Flow Cytometer (Thermofisher, Waltham, MA) with approximately 1.0x10^6^ events collected per sample. Exact counts of cells were determined by the NxT Flow Cytometer software based upon volumetric calculation. Data was normalized to the amount of acquired events and analyzed using FlowJo (Treestar, Ashland, OR) software. LDGs were identified as CD45^+^/CD11b^+^/CD16^+^/CD14^-^/CD15^+^ cells. NET forming LDGs were identified as CD45^+^/CD11b^+^/CD16^+^/CD14^-^/CD15^+^/MPO^+^/CitH3^+^ cells. Platelet bound LDS were identified as CD45^+^/CD11b^+^/CD16^+^/CD14^-^/CD15^+^/MPO^+^/CitH3^+^/CD41^+^ cells. The activation of platelet bound neutrophils was determined by the presence or absence of CD62P (P-selectin).

### Statistics

This is a cross sectional study comparing PPASC, NRS, and NP participants. Flow cytometry results were analyzed using Mann Whitney-U test with a 95% confidence interval for between groups comparisons. Spearman correlations were performed for between variable associations with a linear regression model for multi-variate analysis. A p value < 0.05 was considered statistically significant for all tests. Statistical analysis was performed using Prism 9 (Graph Pad, San Diego, CA).

## Results

### Circulating LDGs display increased frequency of maturation/activation

To understand the impact of long-term COVID-19 on LDGs and LDG dysregulation in pulmonary PASC, we selected samples from COVID-19 convalescents with pulmonary symptoms (PPASC group [PPASC], n=12), COVID-19 convalescents with no residual symptoms (recovered group [NRS], n=10). A comparator group of HIV-seronegative controls from a previous HIV cohort study was used as a SARS-CoV-2 naïve control (naïve group [NP], n=12). All groups were matched for age and gender. The baseline participant characteristics are displayed in **Table 1**. The median age of participants was 57, 55, and 54.5 years for NP, NRS, and PPASC participants, respectively. The majority of participants were male (84.4% of all participants) and the prevalence of pre-existing conditions, except for diabetes, was similar between groups. PPASC participants had a significant increase in BMI compared to NP, but not NRS. Months post-infection denotes the length of time from positive SARS-CoV-2 PCR test following symptomatic disease to study enrollment, and these values did not differ between NP and PPASC groups (**Table 1**). The majority (>70%) of NRS and PPASC participants were fully vaccinated for SARS-CoV-2 (minimum of two doses of Moderna or Pfizer-BioNTech, or one dose of the J&J vaccines). Of our participants vaccinated, 100% completed their full course before SARS-CoV-2 infection within the NRS group, and 90% completed their full course prior to SARS-CoV-2 infection within the PPASC group. Within the PPASC group, 10 of our 12 participants (83.3%) had extra-pulmonary symptoms, the most prominent of which was fatigue (**Table 1**). Within the PPASC participants, 25% were at some point hospitalized due to COVID-19 disease severity (**Table 1**).

**Table 1:** Demographics and Pre-existing Conditions of naive participants (NP), no residual symptoms (NRS), and pulmonary PASC (PPASC) participants.

			NP (n = 12)	NRS (n = 10)	PPASC (n = 12)
**Demographics**	Age (years)		57.0 [53.3-63.5]	55.0 [43.0-60.0]	54.5 [49.8-58.0]
	Male (n, %)		10 (83.3)	7 (70.0)	10 (83.3)
	Caucasian (n, %)		9 (75.0)	3 (30.0)	4 (33.3)
	BMI (kg/m^2^)		27.8 [23.9-29.0]	26.3 [20.6-33.0]	30.8 [26.2-34.1]*
	Months post-Infection		0 [0]	5 [1.5-9.5]	6 [4-14]
	Hospitalized for COVID-19 (n, %)		–	0 (0)	4 (25.0)
**Pre-Existing Conditions**	Diabetes (n, %)		1 (8.3)	1 (10.0)	4 (33.3)
	Hypertension (n, %)		4 (33.3)	2 (20.0)	5 (41.7)
	History of Smoking (n, %)		6 (50.0)	3 (30.0)	4 (33.3)
	COPD (n, %)		0 (0)	0 (0)	0 (0)
	Asthma (n, %)		3 (25.0)	1 (10.0)	2 (16.7)
**COVID Vaccination Status**	Vaccinated for COVID-19 (n/%)		–	7 (70.0)	10 (83.3)
	Vaccinated before COVID-19 Diagnosis (n, %)		–	7 (70.0)	9 (75.0)
**Long-COVID Symptoms**	Non-Specific	Fatigue	–	–	7 (58.3)
		Hair Loss	–	–	1 (8.3)
		Insomnia	–	–	1 (8.3)
	Neuropsychiatric	Anxiety/Depression	–	–	2 (16.7)
		Brain Fog	–	–	3 (25.0)
	Pain	Headache	–	–	3 (25.0)
		Joint Pain	–	–	2 (16.7)
	Oropharyngeal	Loss of taste/smell	–	–	2 (16.7)

Values presented as [median, Q1-Q3] unless otherwise stated. *p < .05 compared to NP.

**Figure 1:**
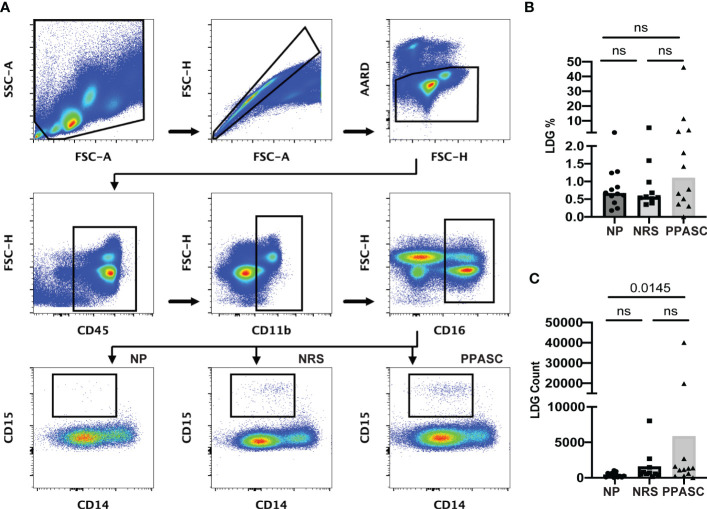
Circulating low-density granulocytes (LDGs) flow cytometry gating strategy. Representative gating for LDGs identified by CD15 expression after gating on CD45^+^CD11b^+^CD16^+^CD14^-^ cells. Boxed regions within scatter plot represents LDGs within NP (n = 12), NRS (n = 10), and PPASC (n = 12) groups **(A)**. Percentage of LDG **(B)** and total count of LDGs in NP, RC, and PPASC groups **(C)**. Graphs are shown as bar with scatter dot plots. Comparisons between groups were performed using Mann-Whitney U test. ns, non-significant, p > 0.05.

LDGs were identified as CD45^+^/CD11b^+^/CD14^-^/CD16^+^/CD15^+^ cells (**Figure 1A**). The PPASC group demonstrated an increasing trend in the percentage of LDGs (1.1% [0.40-3.50%]) (**Figure 1B**) and significantly elevated LDG counts (1243 [358-2421]) as compared to NP (0.67% [0.45-1.14%]; 372 [95-652], respectively) (**Figure 1C**). Interestingly, LDG counts were elevated, albeit not significantly, in NRS than NP although their frequencies were comparable to NP, suggesting that there is preferential expansion of LDG populations in NRS.

**Figure 2:**
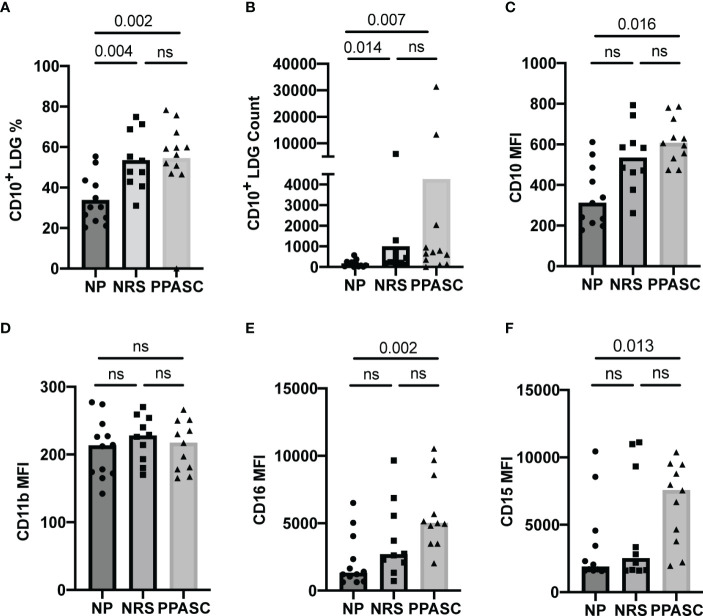
LDG display a mature phenotype in COVID-19 convalescents. Mature LDGs were identified by their CD10 expression. The percentage **(A)** and total number **(B)** of CD10^+^ cells in the LDG fraction were compared across three groups. Analysis of surface marker expression on circulating LDGs and median fluorescence intensity (MFI) of CD10 **(C)**, CD11b **(D)**, CD16 **(E)**, and CD15 **(F)** on LDGs in NP, NRS, and PPASC groups. Graphs are shown as bar with scatter dot plots. Comparisons between groups were performed using Mann-Whitney U test. ns, non-significant, p > 0.05.

Frequencies of LDGs positive for CD10, known as a mature granulocyte marker, were significantly increased in NRS and PPASC as compared to NP (**Figure 2A**). CD10^+^ LDG counts also were significantly increased in PPASC (p=0.007) and NRS (p=0.014) compared to NP (684 [182-1766], 337 [190-913], 105 [31-243], respectively) (**Figure 2B**).

**Figure 3:**
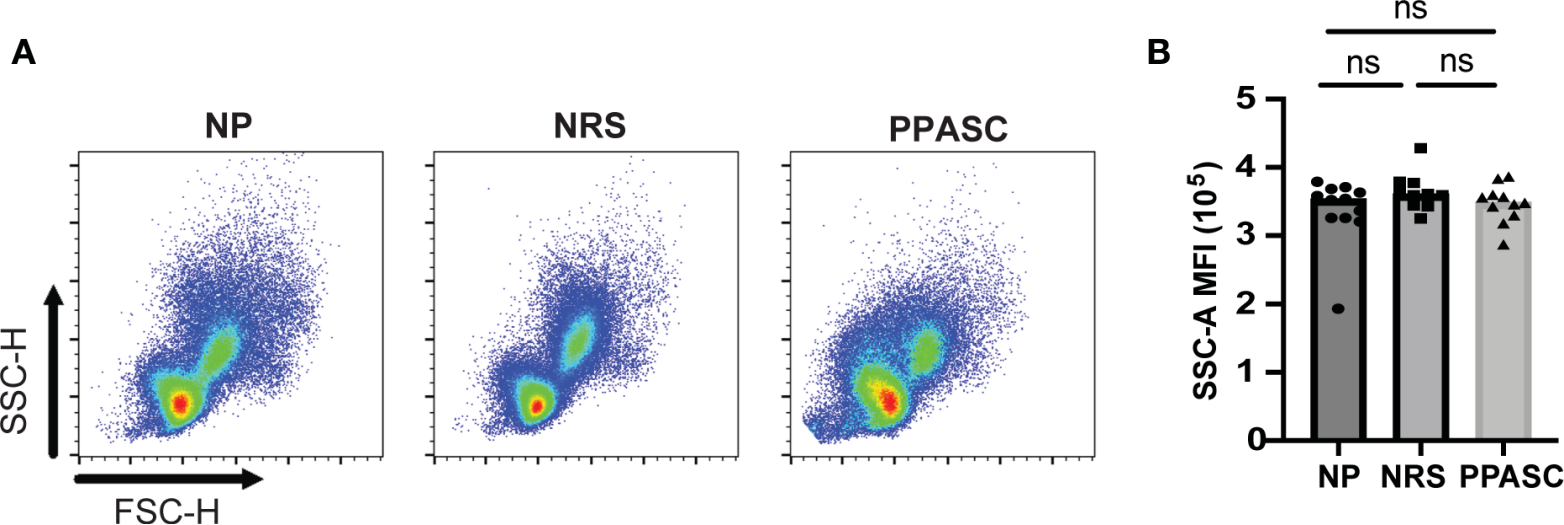
Granularity of LDGs after SARS-CoV-2 Infection. Morphology (size and granularity) of LDGs were analyzed by flow cytometry. Gating strategy for identification of granularity on LDG after gating on CD45^+^CD11b^+^CD16^+^CD14^-^CD15^+^cells **(A)**. Mean granularity of LDGs based on sideward scatter/area of intensity (SSC-A) in NP, NRS, and PPASC **(B)**. Graphs are shown as bar with scatter dot plots. Comparisons between groups were performed using Mann-Whitney U test. ns, non-significant, p > 0.05.

Both LDGs from NRS and PPASC displayed higher median fluorescence intensity (MFI) of CD10 than NP (**Figure 2C**). Indeed, MFI expression of CD15 and CD16 per granulocyte was increased in PPASC compared to NP, although there was a rising trend of increased expression within NRS (**Figures 2E, F** **)**. Next, we assessed the activation status of LDGs by determining CD11b and MPO expression and granularity. There was no difference in MFI expression of CD11b and granularity between the three groups (**Figures 2D**, **3**). Taken together, our data indicate that circulating LDGs display increased frequency of maturation and their levels remain elevated for many months after infection, even within patients who recovered from COVID-19 with no residual symptoms.

### PPASC exhibits elevated levels of CD16^hi^ LDGs in circulation

**Figure 4:**
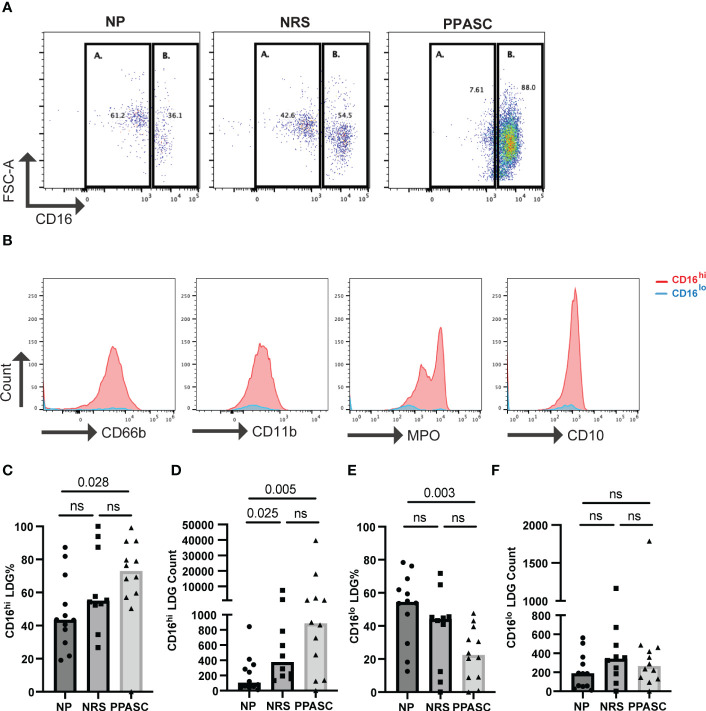
Representative dot plot showing LDG populations in NP, NRS, and PPASC separated into CD16^lo^ **(A)** and CD16^hi^ **(B)** populations **(A)** Representative histograms showing expression of CD66b, CD11b, MPO, and CD10 in LDG populations from PPASC participants according to CD16^lo^ (blue) and CD16^hi^ (red) delineation **(B)** CD16^hi^ LDG % in NP, NRS, and PPASC groups **(C)** CD16^hi^ LDG counts in NP, NRS, and PPASC groups **(D)** CD16^lo^ LDG % in NP, NRS, and PPASC groups **(E)** CD16^lo^ LDG count in NP, NRS, and PPASC groups **(F)** Mann-Whitney U Test, ns, non-significant, p > 0.05.

Recent studies have identified different LDG subsets based on the relative expression levels of CD16 in COVID-19 patients (23–26). Our data indicated that LDG from NRS and PPASC exhibited more mature neutrophils, compared to LDG from NP. Indeed, the trend of higher CD16 MFI expression was detected on LDG from PPASC, which led us to further examine if a specific LDG subset (based on CD16 expression) is increased in PPASC. Similar to previous observations, we found two LDG subsets (CD16^lo^ and CD16^hi^) within PBMC (**Figure 4A**). CD16^hi^ LDG from PPASC displayed a mature neutrophil phenotype denoted by CD10 expression (**Figure 4B**). Notably, increased frequencies and numbers of CD16^hi^ LDG appeared in PPASC, compared with NRS and NP (**Figures 4A, C–F**). These data suggest that higher CD16 MFI expression in PPASC is related to expansion of CD16^hi^ LDG.

### LDGs from PPASC demonstrate an enhanced ability to form NETs

**Figure 5:**
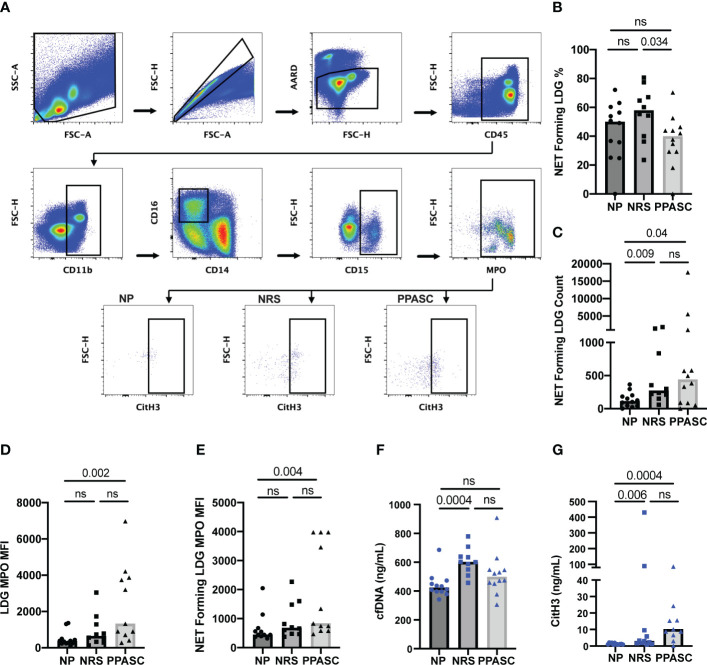
Characterization of NET formation in LDGs. Whole PBMC were gated on side scatter area (SSC-A) and forward scatter area (FSC-A) to gate out debris. Forward scatter height (FSC-H) and FSC-A gated out doublets followed by a viability gate using LIVE/DEAD stain and FSC-A. Leukocytes were selected by CD45 expression and CD11b^+^CD14^-^CD16^+^ fractions were finally identified for LDG based on CD15 expression. NETs were defined as MPO and CitH3 double positive events within the LDG populations for NP, NRS, and PPASC **(A)**. The percentage **(B)** and total number **(C)** of NET forming cells in the LDG fraction was compared across three groups. MFI of MPO on LDGs **(D)** and NET forming LDGs **(E)** in NP, NRS, and PPASC groups. cfDNA amounts measured *via* ELISA in NP, NRS, and PPASC groups **(F)**. CitH3 amounts measured *via* ELISA in NP, NRS, and PPASC groups **(G)**. Graphs are shown as bar with scatter dot plots. Comparisons between groups were performed using Mann-Whitney U test. ns, non-significant, p > 0.05.

NET forming LDGs were identified as double positive for citrullinated histone H3 (CitH3) and MPO by flow cytometry (**Figure 5A**). Although the frequency of LDGs able to form NETs was decreased in PPASC, the numbers of NET forming LDGs were moderately increased in both NRS and PPASC, compared to NP (**Figures 5B, C** **)**. COVID-19 convalescents showed increased LDGs displaying mature phenotype. Indeed, MFI of MPO expression on LDGs was higher in these individuals (**Figure 5D**), suggesting that COVID-19 convalescents may possess more abundant granules within the neutrophils. LDGs have been proposed as the main source of NETs due to their enhanced capacity of spontaneous NET release in autoimmune and inflammatory diseases. In addition, MPO expression levels derived from NET forming LDGs in PPASC were increased compared to NP, potentially indicating increased neutrophil activation in PPASC (**Figure 5E**). To verify whether plasma of COVID-19 convalescents contained increased concentrations of NETs as a consequence of increased numbers of circulating LDGs with enhanced ability to release NETs, we quantified plasma level of cell-free DNA (cfDNA) and CitH3. As shown in **Figures 5F, G**, we observed elevated cfDNA in NRS compared to NP, and elevated H3cit levels in NRS and PPASC, compared to NP. These results are the first to demonstrate an increase in NET forming LDGs and plasma NET levels in convalescent SARS-CoV-2 infected individuals following COVID-19.

### Platelets form aggregates with LDGs in COVID-19 infected individuals

**Figure 6:**
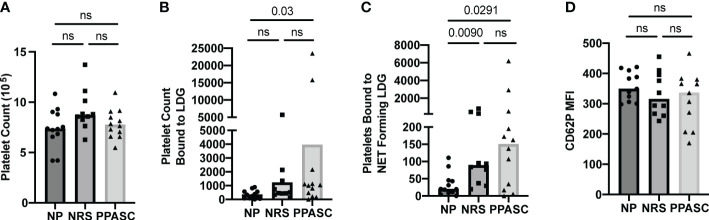
Total number of platelets **(A)**, bound to LDGs **(B)**, bound to NET forming LDGs **(C)**, and MFI of CD62P on platelet bound to NET forming LDGs in NP, NRS, and PPASC **(D)**. Graphs are shown as bar with scatter dot plots. Comparisons between groups were performed using Mann-Whitney U test. ns, non-significant, p > 0.05.

After observing increased NET forming LDGs in both PPASC and NRS individuals, we hypothesized that an increase in NETs would favor PNA. We identified PNA’s through flow cytometry, gating first on LDG populations and then gating for markers of platelets (CD41) and their activation (CD62P). As platelet morphology would traditionally gate them out upon initial forward and side scatter gating, identification of platelets after the LDG gate suggests PNA formation. Although the number of platelets was no different between three groups (**Figure 6A**), we observed a significant increase in the numbers of platelets bound to LDGs within the PPASC group (**Figure 6B**). As NETs have been demonstrated to provide scaffolding for platelet adhesion, activation, and aggregation, we examined platelet populations bound specifically to NET forming LDG populations. As expected, COVID-19 convalescents showed an increase in platelet binding to NET forming LDGs, which is more prominent in COVID-19 convalescents who had residual pulmonary symptoms (**Figure 6C**). To determine if activated platelet facilitate PNA in COVID-19, we examined CD62P (P-selectin) expression on platelets bound to NET forming LDGs. MFI expression of P-selectin was not different between three groups, suggesting that PNA is independent of P-selectin expression on platelets (**Figure 6D**).

## Discussion

A comprehensive understanding of the host immune response and the immunopathological characteristics in COVID-19 individuals with PASC is important for developing therapeutic treatments to target lingering symptoms. Elevated levels of LDG and their phenotypic activation are thought to play an important role in disease severity in COVID-19 patients (12). However, their concentration in circulation and functional contributions to long-term effects of COVID-19 are currently unknown. In this study, we analyzed a cross-sectional LDG population and compared circulating LDG levels in patients with PPASC and with no residual symptoms to individuals naïve to SARS-CoV-2. We also investigated the phenotypic and functional alteration of LDGs in the study participants. To the best of our knowledge, there is currently no report regarding the long-term effect of SARS-CoV-2 on LDGs in COVID-19 individuals and this is the first time it has been shown that circulating LDGs remain elevated during PPASC onset, following SARS-CoV-2 infection. Even more remarkable was the observation that profound increases in CD10^+^, mature LDG numbers were observed in both NRS and PPASC groups. Specifically, the increased CD10^+^ LDG counts in these individuals were reflected as a significant increase in the proportion of CD10^+^ cells within the LDG population.

Studies have shown that SARS-CoV-2 infection leads to alterations of the frequency and functional phenotypes of neutrophils. Elevated immature granulocytes in the blood of patients with severe COVID-19 correlates with the disease severity (27, 28). Silvin et al. (2020) found that severe COVID-19 led to profound increases in immature myeloid cells, such as HLA-DR^low^ monocytes and CD10^low^/CD101^+/-^ neutrophils. These data suggested that MDSC expansion and elevated calprotectin could therefore predict the severity of the outcome of COVID-19. However, only 25% of PPASC individuals were hospitalized, and we do not have results that support neutrophil maturation status in PPASC individuals correlating to hospitalization due to SARS-CoV-2 infection, likely due to our small sample size. In addition, it is still largely unknown whether the immunosuppressive activities of LDG are altered in PPASC, and therefore contribute to long-term pulmonary sequelae. Further studies will be required to dissect the immunosuppressive nature of granulocytic MDSC between NRS and PPASC.

In Frishberg et al., (29), integrating blood transcriptomics into a pseudotime modeling, they found that severe COVID-19 patients were identifiable *via* the high abundance of mature neutrophils. In addition, mature neutrophils subsets, such as MME^+^, IFIT^+^, CXCL8^+^ neutrophils were negatively associated with low pseudotime, therefore reflecting disease severity. Further analysis showed that mature neutrophils, but not T cell or B cell subsets, were strongly inversely correlated with recovery pseudotime, suggesting that severe COVID-19 patients who have persistent elevation in mature neutrophil levels over time is predictive for unfavorable disease outcomes. In line with this finding, George et al., 2022 observed increases in systemic neutrophil counts and MPO expression, primarily in severe COVID-19 survivors diagnosed interstitial lung disease in comparison with resolved individuals (30). Furthermore, MPO levels were positively correlated with disease extent visualized by CT scan and negatively associated with TLCO%. In our PPASC cohort, we observed no significant associations between DLCOc% and LDG, MPO^+^ LDG, and NET forming LDGs (data not shown). Studies have shown that PFTs in severe COVID-19 survivor with lung abnormalities after hospital discharge experience significant pulmonary sequelae and were more likely to have persistent pulmonary symptoms (31, 32). This is likely a result of our PPASC participants experiencing less severe COVID-19, with only 25% ever hospitalized during disease. LDG dysregulation per se may not be a factor contributing to pulmonary tissue damage in case of COVID convalescent who underwent a mild disease course. Further studies are necessary to better characterize whether the severity of COVID-19 drives pulmonary damages differentially through modulation of neutrophils’ functionalities.

Additional studies have highlighted that increased numbers of immature granulocytes in the blood of patients with severe COVID-19 correlates with the disease severity (28, 33) reported that both CD10^-^ (immature) and CD10^+^ (mature) LDG population were significantly higher in severe/critical COVID-19 patients. In context of COVID-19, LDGs expressing CD10^low^ resemble myeloid-derived suppressor cells (27, 34), in contrast mature LDGs with higher CD10 and CD16 expression are more prone to form NETs (35). In line with previous findings (29), LDGs from COVID-19 convalescents demonstrated more mature granulocytes with higher expression of CD10, CD16, and MPO, in combination with enhanced NET production.

We futher found that the proportions of CD16^hi^ LDGs were significantly higher in PPASC than NP, in contrast to the reduced proportions of CD16^lo^ LDGs in the PPASC group. As previously described, neutrophil morphology in COVID-19 patients vary between mature segmented and immature round nuclei (25). Moreover, a majority of CD16^int^ neutrophils contained band-shaped nucleus, but CD16^hi^ neutrophils were bilobed rather than hypersegmented, a similar morphology observed in other severe infections (36). These findings suggest the possibility of morphological abnormalities in COVID-19 individuals even in mature neturophils. Although morphological analysis of neutrophils was not performed in this study, CD16^hi^ LDGs showed enhanced neutrophil activation and displayed higher MFI of CD66b, CD11b, and MPO. Our findings suggest that the activation of of mature LDGs and their high affinity for platelet aggregation may contribute to thrombosis among individuals with PPASC. The maturation status of LDG during long-term symptoms associated with post-SARS-CoV-2 infection should be of prominent interest for understanding the association of LDGs with PPASC onset and persistence.

Our study was limited in multiple ways. Our sample size was small. The length of time following COVID-19 confirmation and enrollment was variable, ranging from 1 to 10 months post-infection. This variability in time of sample collection may have influenced LDG population characteristics. Interestingly, no observed correlations between length of time post-infection and LDG population levels was observed (data not shown). Thus, a longitudinal evaluation of neutrophil dynamics and functional properties after COVID-19 infection should be carried out to determine whether neutrophil dysfunction is associated with the clinical outcome of respiratory failure. No significant correlations of LDG populations and DLCOc% were observed, suggesting further investigation is warranted (data not shown).

Hematological analysis would provide direct evidence of changes in whole blood cellular composition, however complete blood counts (CBC) were not performed on study participants. As a result, a comparison of our flow cytometric evaluations to clinical CBC counts was not possible. Examination of NET formation in isolated neutrophils *ex vivo* would provide evidence that increased NETs in COVID convalescents likely a result of neutrophils’ increasingly activated state. However, we obtained cryopreserved study participant specimens, thus freshly drawn blood samples were not available when we conducted this study. Our study did not contain analysis into circulating biomarkers for immunosuppression and inflammation related to neutrophil activation. Thus, further analysis is required to understand whether the changes in LDGs correlate with markers for such immune skewings in COVID-19 convalescents. Our examinations yielded evidence that statistical differences between NRS and PPASC groups was not consistently found. This is likely due to small sample size but could also indicate that exposure to SARS-CoV-2 promotes the perturbations examined herein, not necessarily the participants progression in PPASC or recovery with no residual symptoms. Regardless, larger studies with higher statistical power are necessary to delineate such complexities.

In conclusion, our data demonstrate that SARS-CoV-2 infection leads to long-lasting alterations in the peripheral LDG functionality and activation, including enhanced NET formation and platelet aggregation. These alterations may contribute to increased tissue damage and thrombosis following infection with SARS-CoV-2 and suggest that sustained expansion of neutrophils subsets may correlate with prolonged respiratory symptoms. Further studies are needed to determine whether targeting of the neutrophil subset, including LDGs, would be beneficial to prevent the development and persistence of PPASC.

## Data availability statement

The raw data supporting the conclusions of this article will be made available by the authors, without undue reservation within reason.

## Ethics statement

The studies involving human participants were reviewed and approved by University of Hawaii at Manoa Human Studies Institutional Review Board (IRB#: RA-2020-053) and the Queens Medical Center Institutional Review Committee (IRB#: RA-2020-053). The patients/participants provided their written informed consent to participate in this study.

## Author contributions

LD and JP designed the experimental approaches. BJ collected and processed human blood samples and clinical data collection. LD, BJ, NS, and JP conducted the experiments. LD conducted and analyzed flow cytometry data. GD analyzed PFT testing. LD, GD, BJ, NS, DC, and JP analyzed data. LD, NS, and JP prepared figures. LD, GD, BJ, and JP wrote the manuscript. LD, GD, MT, VN, SC, DC, CS, and JP, edited and revised the manuscript. LD and JP approved the final version of the manuscript. All authors contributed to the article and approved the submitted version.

## Supplementary material

Supplementary Table 1 Flow cytometry primary antibody and fluorophore conjugates.
