# Corrigendum: Phenotypic alteration of low-density granulocytes in people with pulmonary post-acute sequelae of SARS-CoV-2 infection

**DOI:** 10.3389/fimmu.2023.1209624

**Published:** 2023-04-28

**Authors:** Logan S. Dean, Gehan Devendra, Boonyanudh Jiyarom, Natalie Subia, Michelle D. Tallquist, Vivek R. Nerurkar, Sandra P. Chang, Dominic C. Chow, Cecilia M. Shikuma, Juwon Park

**Affiliations:** ^1^ Hawaii Center for AIDS, John A. Burns School of Medicine, University of Hawai’i at Manoa, Honolulu, HI, United States; ^2^ Department of Tropical Medicine, Medical Microbiology, and Pharmacology, John A. Burns School Medicine, University of Hawai’i at Manoa, Honolulu, HI, United States; ^3^ Department of Pulmonary and Critical Care, Queen’s Medical Center, Honolulu, HI, United States; ^4^ Department of Medicine, John A. Burns School of Medicine, University of Hawai’i at Manoa, Honolulu, HI, United States; ^5^ Center for Cardiovascular Research, John A. Burns School of Medicine, University of Hawai’i at Mānoa, Honolulu, HI, United States

**Keywords:** SARS-CoV-2, long-COVID, post-acute sequelae of SARS-CoV-2 infection, pulmonary sequelae, low-density granulocyte, neutrophil extracellular traps, platelets, platelet neutrophil aggregates

In the published article, there was an error in the article title. Instead of “Phenotypic alteration of low-density granulocytes in people with pulmonary post-acute sequalae of SARS-CoV-2 infection”, it should be “Phenotypic alteration of low-density granulocytes in people with pulmonary post-acute sequelae of SARS-CoV-2 infection”.

The authors apologize for this error and state that this does not change the scientific conclusions of the article in any way. The original article has been updated.

In the published article, there was an error. The spelling of “sequelae” was incorrect in the second sentence of the Introduction.

A correction has been made to the **Introduction.** The second sentence previously stated: “Although most people with COVID-19 infection recover within a few weeks, almost a third of survivors suffer from persistent symptoms, so called “Long COVID”, otherwise known as Post-Acute Sequalae of SARS-CoV-2 Infection (PASC) (2).”

The corrected sentence appears below:

“Although most people with COVID-19 infection recover within a few weeks, almost a third of survivors suffer from persistent symptoms, so called “Long COVID”, otherwise known as Post-Acute Sequelae of SARS-CoV-2 Infection (PASC) (2).”

The authors apologize for these errors and state that this does not change the scientific conclusions of the article in any way. The original article has been updated.

