# Supplementary material for: Phenotypic alteration of low-density granulocytes in people with pulmonary post-acute sequelae of SARS-CoV-2 infection
Source: Front Immunol. 2022 Dec 15;13:1076724. doi: 10.3389/fimmu.2022.1076724 (PMC9797994; doi:10.3389/fimmu.2022.1076724)
Supplement: Supplementary Table 1 — Flow cytometry primary antibody and fluorophore conjugates. [file Table_1.docx]

**Supplementary Table 1**

| **Antibody Target** | **Conjugate Fluorophore** | **Company** |
| --- | --- | --- |
| CD45 | BV711 | BD Biosciences (East Rutherford, NJ) |
| CD11b | PE-Cy-7 | BioLegend (San Diego, CA) |
| CD14 | BV605 | BioLegend (San Diego, CA) |
| CD16 | BV650 | BioLegend (San Diego, CA) |
| CD15 | FITC | Millipore Sigma (St. Louis, MO) |
| CD10 | PerCP5.5 | R&D Systems (Minneapolis, MN) |
| CD41 | PE-Dazzle | BioLegend (San Diego, CA) |
| CD62p | AF700 | BioLegend (San Diego, CA) |
| CD66b | BV421 | BioLegend (San Diego, CA) |
| MPO | PE | BD Biosciences (East Rutherford, NJ) |
| citH3 | APC | Abcam & Invitrogen (Waltham, MA), respectively |
| Viability | eFluor506 | Invitrogen (Waltham, MA) |
